# Eradication of *Pseudomonas aeruginosa* Persister Cells by Eravacycline

**DOI:** 10.1021/acsinfecdis.4c00349

**Published:** 2024-11-13

**Authors:** Sweta Roy, Zeynep S. Cakmak, Salma Mahmoud, Mahsa Sadeghzadeh, Guirong Wang, Dacheng Ren

**Affiliations:** † Department of Biomedical and Chemical Engineering, Syracuse University, Syracuse, New York 13244, United States; ‡ Department of Surgery, SUNY Upstate Medical University, Syracuse, New York 13210, United States; § Department of Microbiology and Immunology, SUNY Upstate Medical University, Syracuse, New York 13210, United States; ∥ BionInspired Institute, Syracuse University, Syracuse, New York 13244, United States; ⊥ Department of Civil and Environmental Engineering, Syracuse University, Syracuse, New York 13244, United States; # Department of Biology, Syracuse University, Syracuse, New York 13244, United States

**Keywords:** biofilm, bacterial pneumonia model, eravacycline, *P. aeruginosa*, persister, antibiotic

## Abstract

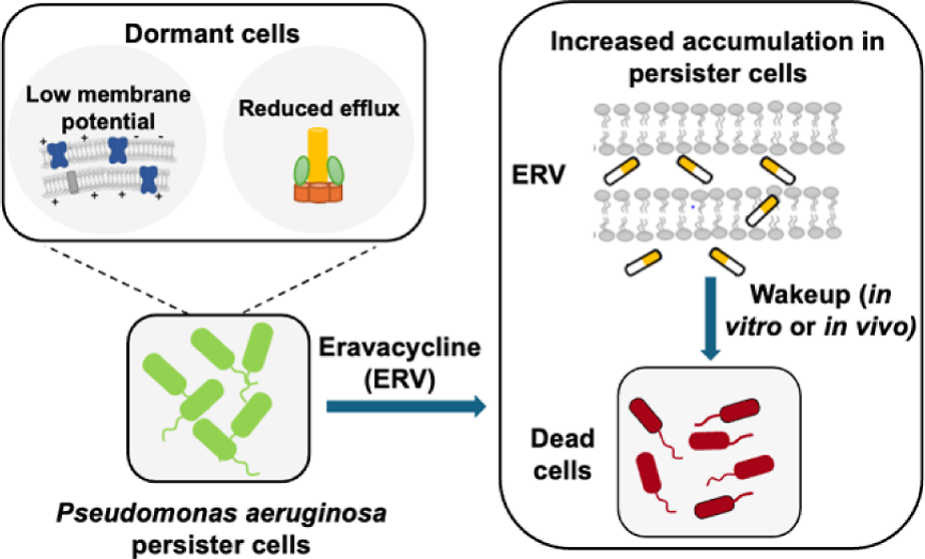

*Pseudomonas aeruginosa* is a leading bacterial pathogen that causes persistent infections. One major reason that antibiotics fail to clear such infections is the presence of a dormant subpopulation called persister cells. To eradicate persister cells, it is important to change drug development from traditional strategies that focus on growth inhibition to the search for new leads that can kill dormant cells. In this study, we demonstrate that eravacycline can effectively accumulate in *P. aeruginosa* persister cells, leading to strong killing during wakeup, including persister cells in both planktonic cultures and biofilms of the wild-type strain and its mucoid mutant. The effects of eravacycline on persister control were further validated *in vivo* using a lung infection model in mice. Collectively, these results demonstrate the possibility to control persister cells of bacterial pathogens by targeting dormancy.

*Pseudomonas aeruginosa* causes major infections that are difficult to eradicate by current antimicrobial therapies. It is a major causative agent of infections associated with surgical wound sites,^1^ bloodstream,^1,2^ orthopedic implants^3^ and community-acquired pneumonia.^4^ It is also a leading cause of fatal episodes of ventilator-associated pneumonia,^5^ catheter-associated urinary tract infections,^1^ and chronic lung infections of cystic fibrosis patients.^6^ A major challenge in controlling persistent infections is the presence of persister cells,^7,8^ which have reduced metabolic activities and are tolerant to most antibitoics.^9−12^ Because conventional antibiotic drug discovery selects leads based on growth inhibition,^13^ dormant persister cells are not targeted in such screens. The failure to sterilize this subpopulation by antibiotic therapy can lead to reoccurrence of infections despite prolonged treatment time.^9,14^

Persister cells are phenotypic variants that do not grow in the presence of an antibiotic; however, the surviving persister cells can regrow and reestablish the population after the antibiotic is withdrawn. Persistence also allows bacteria to mutate over time, contributing to the emergence of resistant strains.^15^ Further challenges arise when treating persister cells in biofilms, which are sessile bacterial communities embedded in an extracellular matrix.^16^ They are commonly formed under stress conditions and are known to harbor a large number of persister cells.^17^ Therefore, effective approaches for eradicating persister cells especially those in biofilms are needed to combat challenging infections.

We recently discovered that the characteristics associated with dormancy can be leveraged to eradicate *Escherichia coli* persister cells.^18^ Compared to normal cells, dormant cells have lower membrane potential and thus reduced proton motive force and decreased drug efflux. Antibiotics that can penetrate bacterial membranes without requiring active transport, can enter persister cells and accumulate if there is a strong binding with the target, causing persister killing during wakeup when the external antibiotics are removed from the environment.^18^ To determine the efficacy of this strategy against other bacterial pathogens, here we characterized the effects of eravacycline (ERV) on *P. aeruginosa* persister cells. ERV is a relatively new antibiotic in the tetracycline family, approved in 2018 by the FDA. It targets the 30S subunit of the ribosome and halts protein synthesis.^19^ Our previous research showed that ERV can effectively target *E. coli* persister cells due to its ability to penetrate *E. coli* lipid membranes and its strong affinity with the 30S ribosomal subunit.^18,20^ Here, we demonstrate that ERV is an effective control agent against *P. aeruginosa*. Specifically, our results reveal that ERV is effective in killing cyanide m-chlorophenylhydrazone (CCCP) induced *P. aeruginosa* persister cells, reducing *P. aeruginosa* biofilms, and killing persister cells in biofilms. We also demonstrate that CCCP-induced persister cells are effectively killed by ERV *in vivo* in a murine model of lung infection when ERV is internalized in the persister cells. In addition to CCCP persisters, ERV was also evaluated against *P. aeruginosa* persisters isolated with other antibiotics. Collectively, we demonstrate that ERV is a potent persister control agent. These results will help guide future drug discovery to better combat persistent infections.

## Results

### ERV is Effective against *P. aeruginosa*Persisters

**Figure 1:**
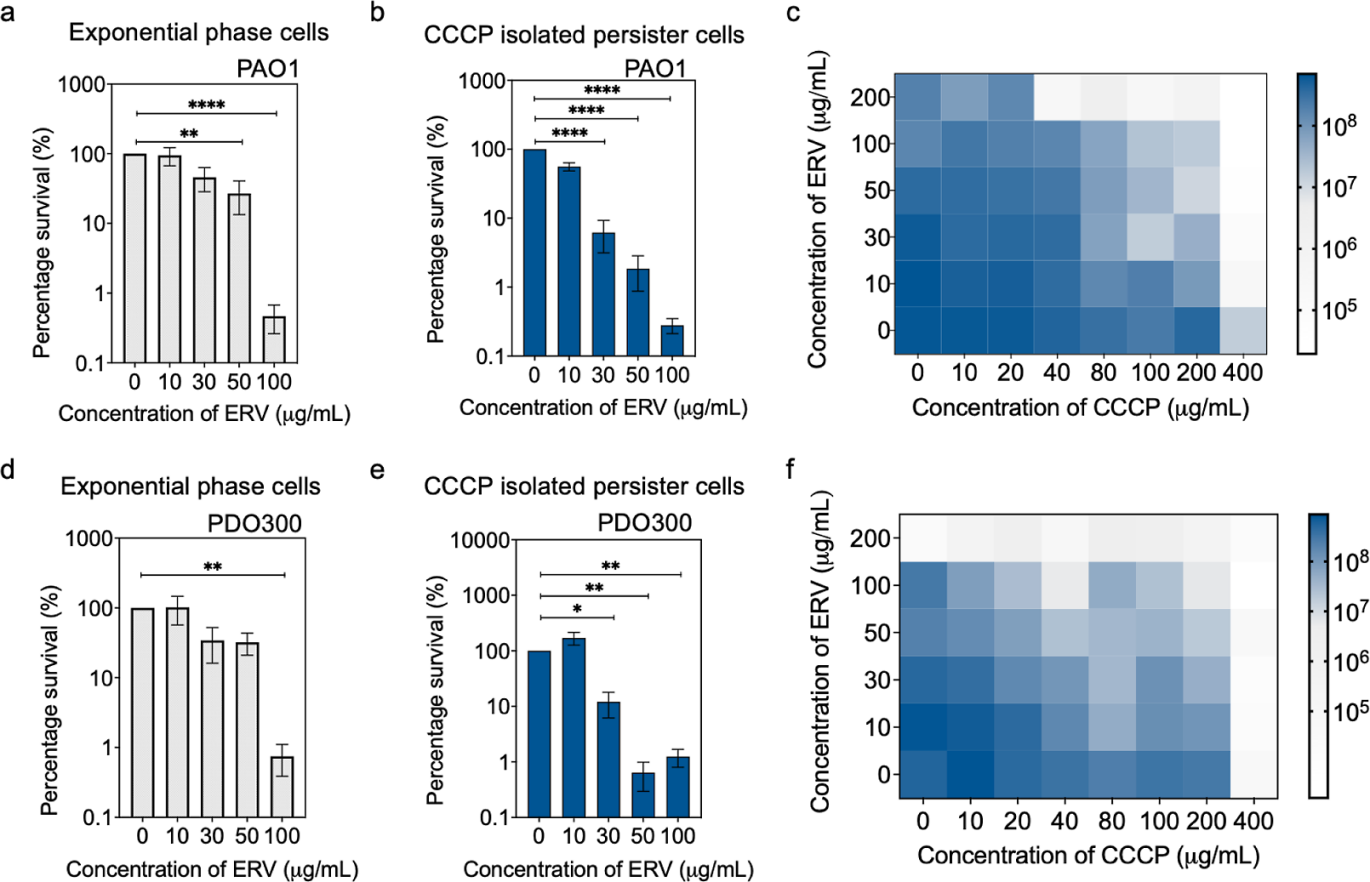
Effects of ERV on normal and persister cells of PAO1 (wildtype) and PDO300 (mucoid mutant). The effects of ERV were compared between normal and persister cells of *P. aeruginosa*. Viability of PAO1 (a) and PDO300 (d) normal cells (exponential phase) were evaluated after treatment with different concentrations of ERV in PBS for 1 h (*n* = 3). Viability of CCCP-isolated persisters of PAO1 (b) and PDO300 (e) were determined after treatment in PBS for 1 h with increasing concentrations of ERV. Means ± SE are shown (*n* = 3). Checkerboard assays of CCCP vs ERV were conducted for PAO1 (c) and PDO300 (f) (*n* = 3). Stationary phase cells were treated with CCCP for 3 h followed by ERV for 1 h.

As a control, we first treated exponential phase *P. aeruginosa* PAO1 (henceforth PAO1) cells (primarily normal cells) with different concentrations of ERV (0–100 μg/mL) in PBS for 1 h. At concentrations of 50 and 100 μg/mL, significant effects were observed with 0.6 (*p* = 0.002) and 2.2 (*p* < 0.0001) logs of killing, respectively (Figure 1a). Next, we tested ERV against PAO1 persisters isolated with 200 μg/mL of CCCP,^21−23^ a condition known to induce a large amount of persister cells in *P. aeruginosa* (∼64% under the condition of this study).^21−23^ After treatment for 1 h in PBS, dose-dependent killing of PAO1 persisters by ERV was observed, e.g., 1.2 logs (*p* < 0.0001), 1.7 logs (*p* < 0.0001) and 2.6 logs (*p* < 0.0001) of killing at concentrations of 30, 50, and 100 μg/mL, respectively (Figure 1b). This aligns with our earlier work on *E. coli* persister cells,^18^ revealing that ERV is more effective in killing persister cells than normal cells of both species.

Checkerboard assays were performed to determine if CCCP and ERV have synergistic or additive effects in PAO1 killing. To accomplish this, stationary phase PAO1 was treated with CCCP (0–400 μg/mL) in LB medium for 3 h followed by ERV (0–200 μg/mL) for 1 h in PBS. Treatment with CCCP alone showed minimal effect against PAO1 (<0.5 log killing with up to 200 μg/mL CCCP). Even at a very high concentration of 400 μg/mL, CCCP had less than 1 log killing (Figure 1c). ERV alone showed moderate killing (<1 log) of stationary phase cells at high concentrations of 100 (74.6 ± 1.6%; *p* = 0.0001) and 200 μg/mL (66.3 ± 5.4%; *p* = 0.0012) (Figure 1c). In comparison, killing by ERV increased significantly by pretreatment with CCCP. For example, treatment with 100 μg/mL of CCCP followed by 100 μg/mL of ERA led to 1.2 logs of killing; and 400 μg/mL of CCCP followed by 100 μg/mL of ERA led to 3.1 logs of killing. This indicates that CCCP induced persister formation rendered the population more susceptible to ERV, which is more effective in killing persisters than normal cells as shown in Figure 1c above. We next evaluated if there is synergistic effect between CCCP and ERV that allowed for this enhanced killing. The IC_50_ (concentration for 50% killing) was found to be 74.4 for CCCP alone, 9.7 for ERV alone, and 3.1 μg/mL for concurrent treatment. Thus, the drug combination index (CI) was found to be 0.4, demonstrating a strong synergy in PAO1 killing.

### Effects of ERV against Normal and Persister Cells of the Mucoid Strain PD0300

During chronic infections, *P. aeruginosa* commonly acquires mutations that lead to mucoid conversion. This enhances its resistance to antibiotics and facilitates evasion of the host defense. The *mucA* gene is frequently associated with this conversion and encodes for an antisigma factor of AlgT.^1,6,24^ To assess the potential of this persister control strategy in controlling more challenging mucoid strains, we further tested the effects of ERV on the normal and persister cells of the isogenic mutant PDO300 (*mucA22*).^25^ Similar to the results of the wild-type PAO1, significant killing of PDO300 exponential phase cells only occurred at a high concentration of 100 μg/mL (by 2.0 logs; *p* = 0.004; Figure 1d). Similar to PAO1 results, CCCP persisters of PDO300 were also found more sensitive to ERV than normal cells. Specifically, killing of persisters was 0.9 log (*p* = 0.01), 2.2 logs (*p* = 0.004), and 2.0 logs (*p* = 0.004) after treatment with ERV at 30, 50, and 100 μg/mL, respectively (Figure 1e). A checkerboard assay was also performed for CCCP-induced persisters of PDO300. Like PAO1, CCCP had minimal effects on the normal cells of PDO300. Increasing CCCP concentration led to a larger persister population and as expected, and more killing of PDO300 by ERV (Figure 1f). For example, treatment with 100 μg/mL of CCCP followed by 100 μg/mL of ERA led to 1.1 logs of killing; and 400 μg/mL of CCCP followed by 100 μg/mL of ERA showed 1.6 logs of killing. Based on these results, CCCP and ERV exhibited synergistic effects in PDO300 killing with a CI value of 0.4. Specifically, the IC_50_ values were 220.9 for CCCP alone, 6.3 for ERV alone, and 2.5 μg/mL for concurrent treatment. In summary, persister cells of both the wild-type PAO1 and its mucoid mutant are more sensitive to ERV than their counterparts of normal cells.

### Effects of ERV against *P. aeruginosa* Persisters Isolated with Antibiotics

**Figure 2:**
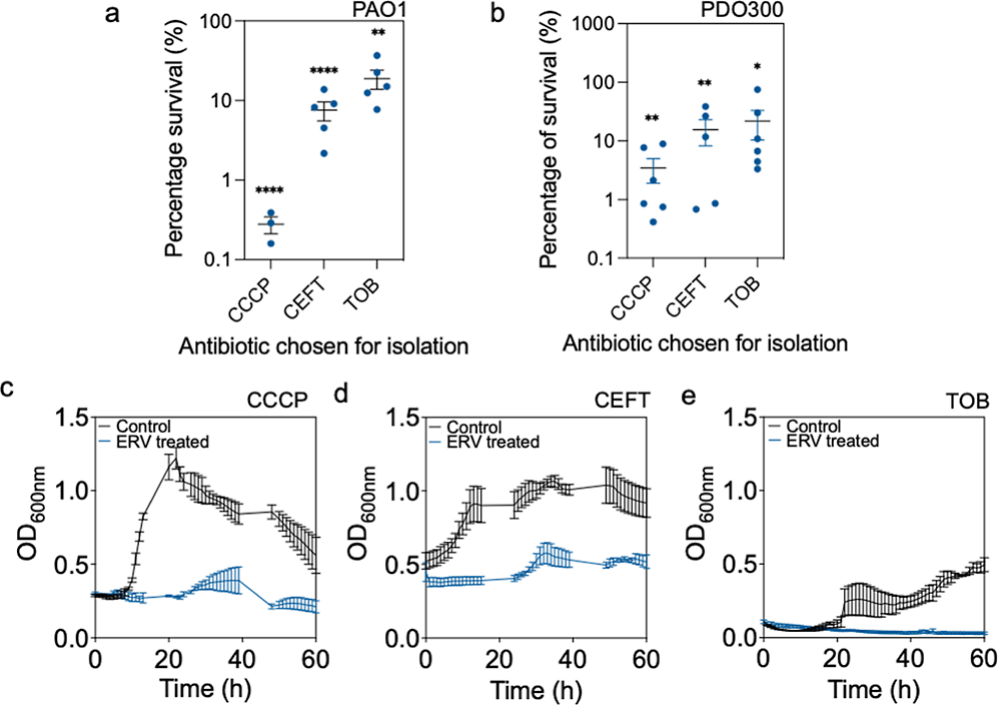
ERV treatment of persister cells isolated with different antibiotics. The effects of ERV were evaluated on *P. aeruginosa* persister cells isolated with different classes of antibiotics. (a,b) Viability of PAO1 (a) and PDO300 (b) persisters isolated with CCCP, CEFT, or TOB after treatment with 100 μg/mL of ERV. Means ± SE are shown (*n* ≥ 3). (c–e) Resuscitation of CCCP (c), CEFT (d), and TOB (e) persisters of PAO1 after treatment with ERV (blue) vs untreated control (black). Means ± SE are shown (*n* = 3).

To corroborate the results of CCCP induced persisters, we further tested the effects of ERV on persister cells isolated with other agents. Antibiotics with different targets were tested including ceftazidime (CEFT) that blocks bacterial cell wall synthesis, and tobramycin (TOB) that halts protein synthesis. Both CEFT and TOB isolated PAO1 persisters were sensitive to ERV with 93.1 ± 0.8% (*p* < 0.0001) and 79.3 ± 5.4% (*p* = 0.0002) killing, respectively, when treated with 100 μg/mL of ERV (Figure 2a). For PDO300, ERV killed 84.4 ± 8.5% (*p* = 0.008) of CEFT-isolated persister cells and 84.9 ± 3.6% (*p* = 0.04) of TOB-isolated persister cells (Figure 2b).

To further corroborate the results and confirm if the killing occurred during wakeup, we monitored the regrowth of ERV-treated and untreated PAO1 persister cells. After washing and resuspending the cells in LB medium, untreated persister cells resumed growth once nutrient was provided. Conversely, persister cells isolated with CEFT, CCCP, or TOB failed to recover after ERV treatment, which indicates that the killing occurred during wake-up (Figure 2c–e). To obtain further insights, postantibiotic effects (PAE) were evaluated for CEFT, CCCP and TOB isolated persister cells and compared with normal cells. PAE represents the period of time when bacterial growth remains suppressed after antibiotic removal.^26^ The PAE value for normal cells was found to be 15.3 ± 0.3 h; while the isolated persister cells of PAO1 all had a PAE value > 60 h (Figure S4). This is because even after 60 h of growth, the treated persister cells did not resuscitate (Figure S4). Overall, these results validate that ERV can penetrate *P. aeruginosa* persister cell membranes and achieve killing during wakeup.

### Effects on *P. aeruginosa*Biofilms

**Figure 3:**
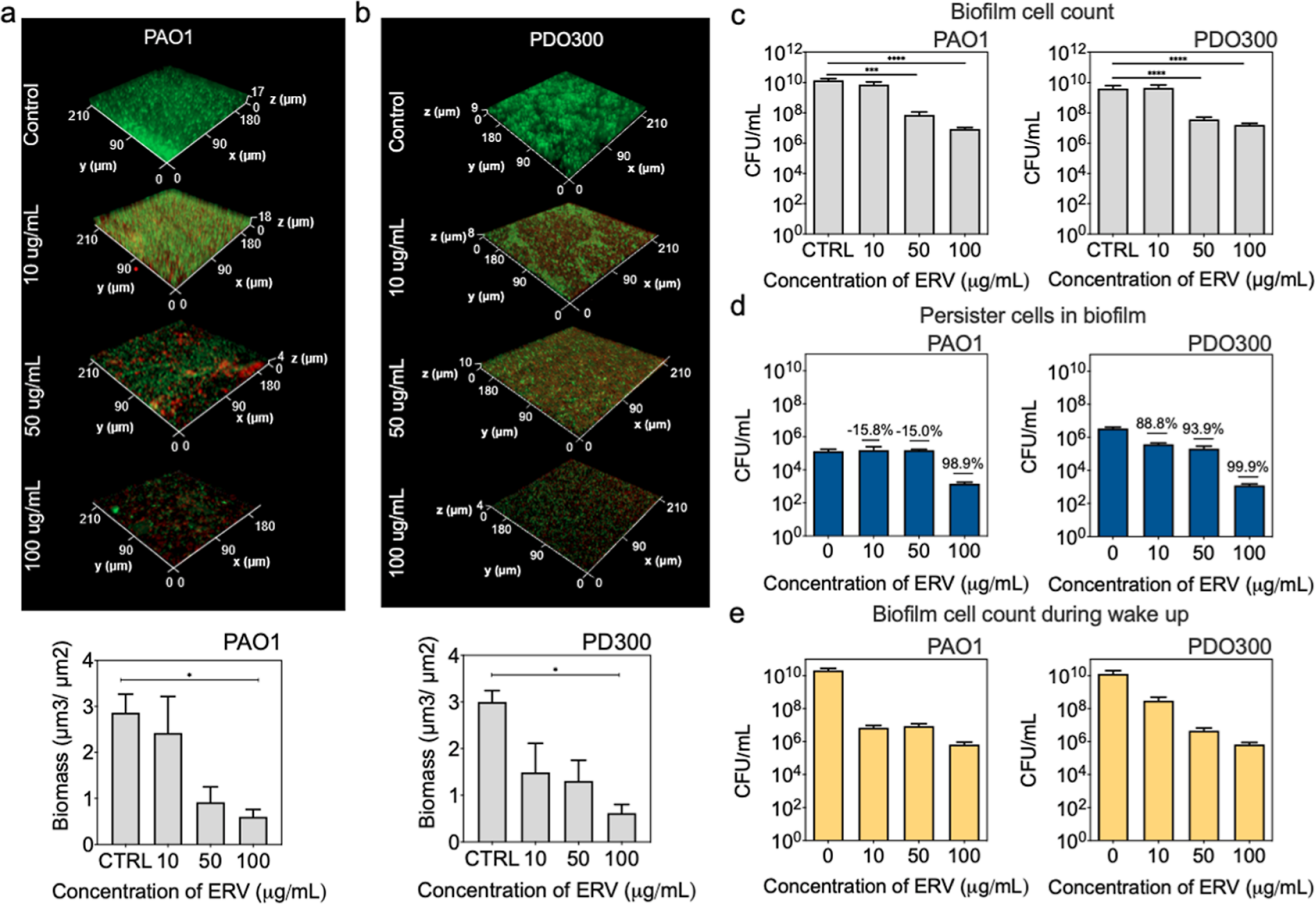
ERV is effective against *P. aeruginosa* biofilm cells and persisters in biofilms. (a,b) Effects of ERV on 48 h biofilms. Biofilms of PAO1 (a) and PDO300 (b) were imaged after ERV treatment. Representative images of biofilms are shown. Biomass was quantified based on SYTO9 signals using COMSTAT after Live/Dead staining. Means ± SE are shown (*n* = 5). (c) Viability of biofilm cells based on CFU for both PAO1 (left) and PDO300 (right). Means ± SE are shown (*n* = 10). (d) Effect on persister cells in biofilms. ERV treated biofilms were further treated with TOB for 4 h to quantify surviving persister cells. Means ± SE are shown (*n* = 3). Percentage represents the killing of persister cells in biofilms compared to the untreated control. (e) Number of PAOl (left) and PDO300 (right) biofilm cells during wakeup (2 h) after treatment with ERV. Means ± SE are shown (*n* = 3).

A major hurdle in controlling cystic fibrosis airway infections caused by *P. aeruginosa* is the formation of biofilms and presence of persister cells in biofilms.^6^ This is partially due to the biofilm matrix, which hinders the penetration of many antibiotics. To understand if ERV is effective against *P. aeruginosa* biofilms and associated persister cells, we tested 48 h biofilms of PAO1 and PDO300 on PDMS (polydimethylsiloxane) surfaces with increasing concentrations of ERV. Dose-dependent reduction of biofilm biomass was obtained, e.g., 0.7 log (*p* = 0.04) by 100 μg/mL ERV (Figure 3a). A similar trend was observed for PDO300 biofilms (Figure 3b). The imaging results were corroborated with CFU assay. Specifically, ERV reduced the number of viable PAO1 biofilm cells by 2.2 logs (*p* = 0.04) and 3 logs (*p* = 0.02), and PDO300 biofilms by 2.1 logs (*p* = 0.002) and 2.4 logs (*p* = 0.002), when treated at 50 and 100 μg/mL, respectively (Figure 3c). The significant decrease in CFU indicates that ERV is able to penetrate biofilms and kill attached cells.

To evaluate the killing of persister cells in biofilms by ERV, the treated biofilm cells were detached and subjected to further treatment with TOB to quantify the number of surviving persisters. This was compared with the control biofilm that was only treated with TOB. No significant persister killing was observed when ERV was added at a concentration of 10 or 50 μg/mL for PAO1 (Figure 3d). However, a 2-log killing was observed when treated at a higher concentration of 100 μg/mL, indicating a threshold concentration for killing. Interestingly, a dose-dependent killing of persister cells in biofilms was observed for PDO300, with 1.0, 1.2, and 3.4 logs of killing observed after treatment with 10, 50, and 100 μg/mL ERV, respectively (Figure 3d).

Like the results of planktonic persister cells, the killing of persister cells in biofilms also occurred during wakeup. After removing the extracellular antibiotic and replenishing the biofilm cells with nutrients, more killing was observed for both PAO1 and PDO300 (Figure 3e). For PAO1, an additional 99.9%, 83.5%, and 91.0% killing was observed for the biofilms treated with 10, 50, and 100 μg/mL of ERV, respectively compared to the initial treatment (before adding LB medium) (Figure 3e vs 3c). For PDO300, an additional 93.2%, 87.8%, and 95.9% killing was observed when the biofilms were treated with 10, 50, and 100 μg/mL of ERV, respectively (Figure 3e vs 3c). In contrast, the control biofilm without ERV treatment showed growth with increasing CFU (∼110% for PAO1 and ∼200% for PDO300; Figure 3e vs 3c). These results indicate that ERV accumulated in persister cells in biofilms leading to killing during wakeup. To further corroborate the results, we quantified the intracellular concentration of ERV in both PAO1 and PDO300 biofilm cells. Using the reporter strain-based bioassay that we previously reported,^18,27^ the intracellular concentrations of ERV in PAO1 and PDO300 biofilm cells were determined to be 21.6 ± 1.9 and 27.5 ± 6.0 μg/mL, respectively, after being treated at 100 μg/mL (Table S1).

Treatment with ERV also killed the detached biofilm cells. For example, ∼10^8^ cells were detached from the biofilm when treated with 100 μg/mL ERV. The detached cells of PAO1 and PDO300 were found to accumulate 23.6 ± 2 and 33.5 ± 0.7 μg/mL ERV, respectively, consistent with the results of attached cells (Table S1). Collectively, these findings revealed that ERV is able to reduce biofilm biomass, penetrate biofilm and kill biofilm cells, and also kill detached cells of both the wild-type PAO1 and its mucoid mutant. ERV also showed strong activities in killing persister cells in biofilms during wakeup.

### In Vivo Validation Using a Murine Lung Infection Model

**Figure 4:**
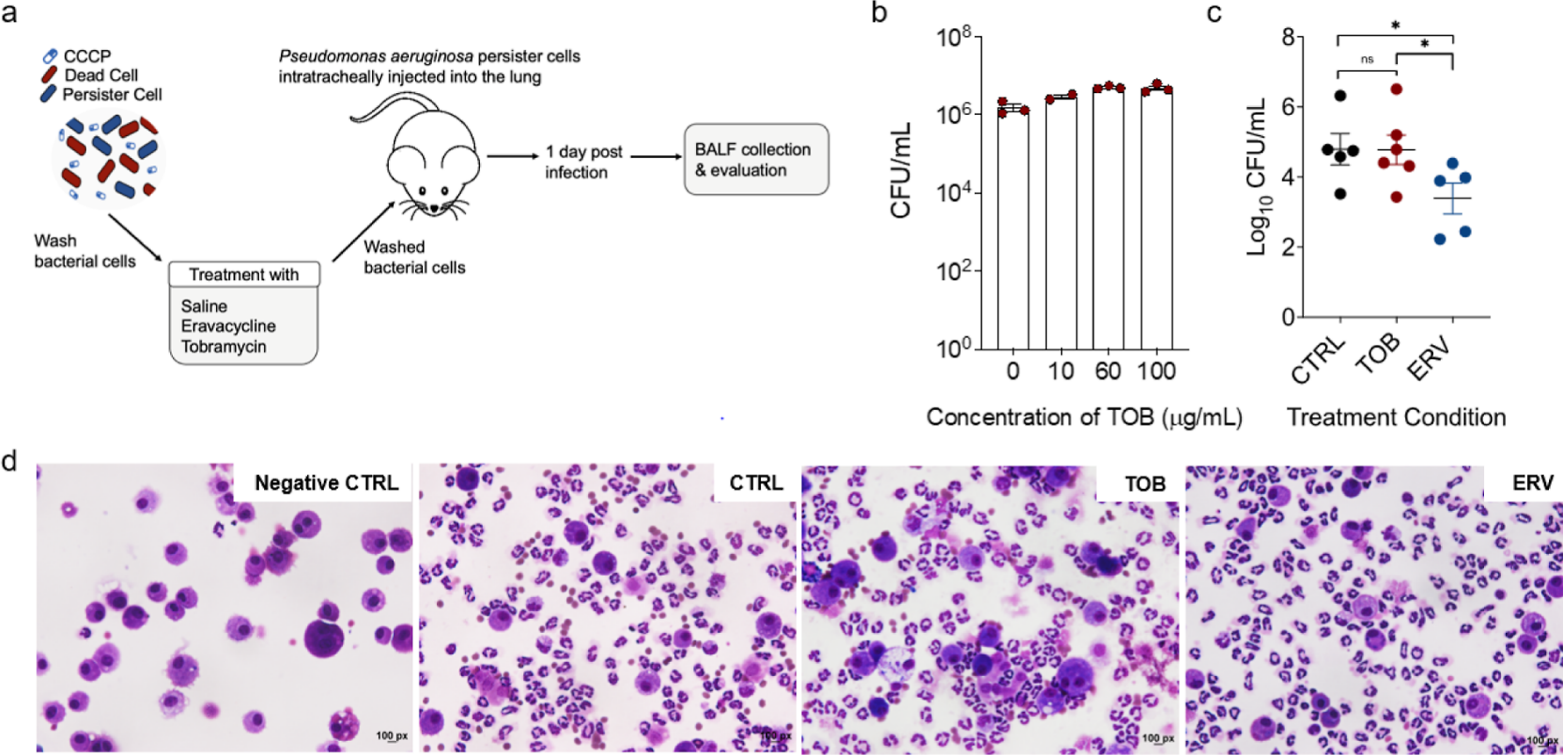
Persister killing *in vivo*. (a) Schematic of persister murine lung infection. Mice were injected with untreated or treated (with 100 μg/mL ERV or 100 μg/mL TOB) CCCP-induced PAO1 persisters cells intratracheally into the lung. (b) CCCP persisters were treated with different concentrations of TOB to determine its effects on *P. aeruginosa* persisters. Samples were incubated for 1 h in PBS. (c) Bacterial load after treatment (24 h post treatment) from BALF was determined based on CFU count. CTRL (*n* = 5) represents mice infected with CCCP induced PAO1 persister cells. TOB (*n* = 6) and ERV (*n* = 5) represent mice infected with treated persister cells (100 μg/mL TOB or ERV). Black lines represent mean ± SE “ns” means not statistical significance; * means *p* < 0.05 after using two-way mixed ANOVA. (d) Representative BALF cytology images of each group after staining with Hema-3 Stain Kit.

With the promising activity of ERV in vitro, we sought to determine the efficiency of ERV *in vivo*. Due to stochastic mechanism of persister formation,^28,29^ low abundance of persister cells in bacterial populations, and rapid reversion of persisters to normal cells with changes in their environment,^9,30^ currently there is no well-established model to directly evaluate persister killing *in vivo*. We thus used a population of isolated persister cells and focused on the test to determine if wakeup *in vivo* provides a sufficient time window for persister killing. To achieve this, we infected the lungs of FVB/N mice with ERV-treated, TOB-treated, and untreated control of CCCP-persisters of PAO1 (Figure 4a). TOB was chosen as a control because it is a commonly used antibiotic for treating *P. aeruginosa* infections; however, it is not effective against PAO1 persisters.^31^ We also validated this *in vitro* in our system by treating CCCP-persisters of PAO1 with different concentrations of TOB (0–100 μg/mL) for 1 h in PBS. No significant killing was observed (Figure 4b).

Mice were intratracheally injected with CCCP persisters of PAO1, TOB-treated persisters, and ERV treated persisters to induce lung infection. At 24 h post inoculation, mice were sacrificed and bronchoalveolar lavage fluid (BALF) was collected to determine bacterial load in the lungs. Consistent with *in vitro* results, ERV-treated persister cells showed a significant decrease in bacterial burden (8.5 × 10^3^ CFU/mL, *p* = 0.02) than TOB-treated persisters (5.8 × 10^5^ CFU/mL). No significant difference was observed between the untreated control and TOB-treated persisters (4.5 × 10^5^ CFU/mL, *p* = 0.89; Figure 4c). These results demonstrate that it is feasible to kill persister cells *in vivo* during wakeup if an appropriate agent is internalized in the bacteria. Both macrophages and neutrophil cells were observed in the infected mice from the BALF compared to the negative control (no bacterial cells were injected), in which only macrophages were detected (Figure 4d). This indicates that bacterial infection in the lungs of mice injected with treated and untreated persister cells can both trigger immune response.

### Eravacycline is Ineffective against Fluoroquinolone Isolated *P. aeruginosa* Persisters

**Figure 5:**
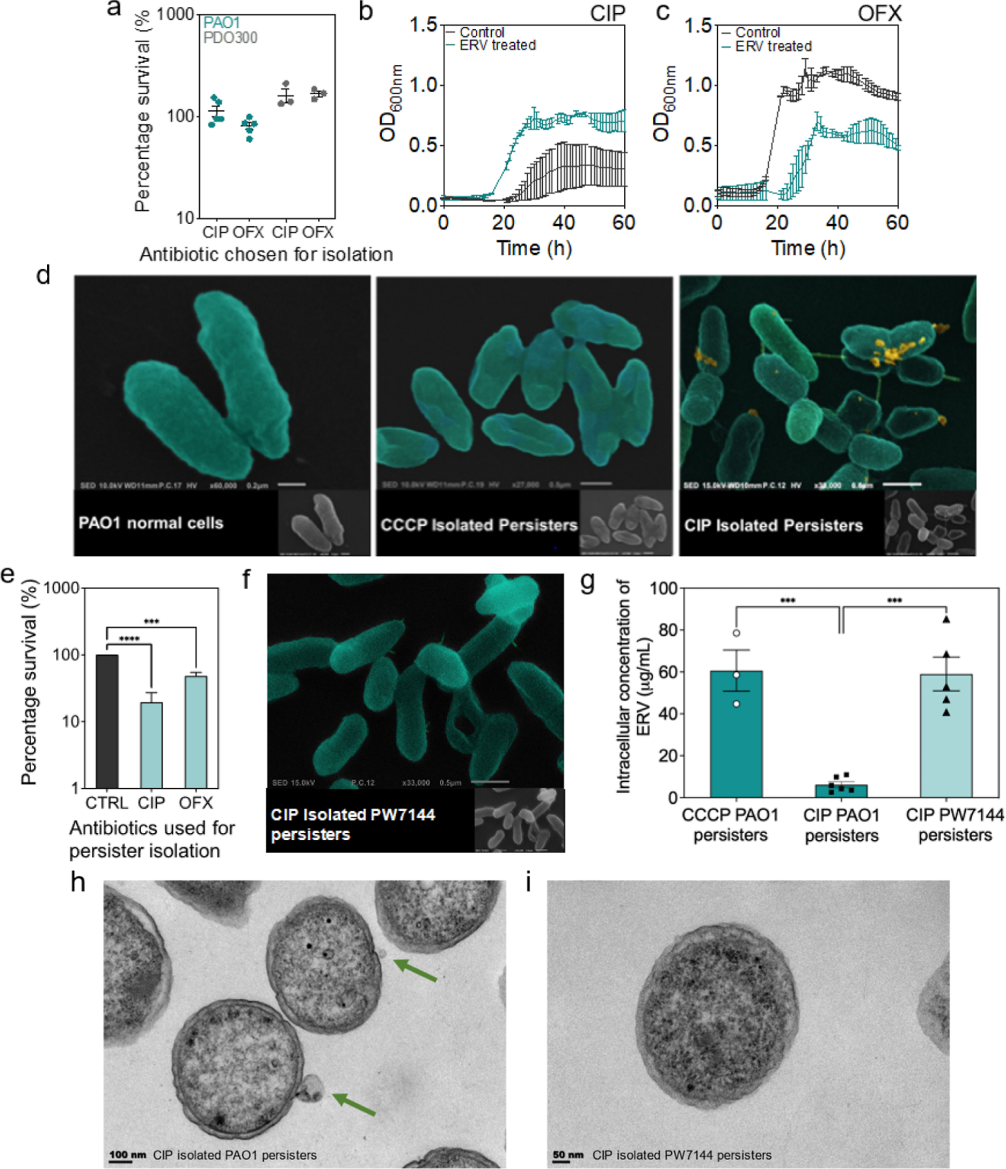
FQ-isolated PAO1 persisters are insusceptible to ERV treatment due to production of OMVs. (a) *P. aeruginosa* persister cells were isolated with FQ antibiotics including CIP and OFX. (a) Viability of PAO1 and PDO300 persisters isolated with CIP and OFX after treatment with ERV at 100 μg/mL. (b) Resuscitation of CIP-isolated PAO1 persisters after ERV treatment (blue) and untreated control (black). (c) Resuscitation of OFX-isolated PAO1 persisters after ERV treatment (blue) and untreated control (black). (d) Representative SEM images of stationary phase PAO1 (control), CCCP-isolated PAO1 persisters, and CIP-isolated PAO1 persisters. Pseudo color was incorporated using MountainsSEM to help illustrate OMVs. The original SEM image is included in the lower right corner of each sample for comparison. Gold outlines indicate the protruding small structures on the membrane. (e) Viability of CIP and OFX isolated PW7144 (*recA* mutant) cells after ERV treatment at 100 μg/mL. Means ± SE are shown (*n* = 3). (f) Representative SEM image of CIP-isolated PW7144 persister cells. (g) Intracellular concentration of ERV in CIP-isolated PAO1 persisters, CCCP-isolated PAO1 persisters, and CIP-isolated PW7144 persisters after treatment at a concentration of 100 μg/mL. Means ± SE are shown (*n* = 3). (h,i) Representative TEM images of CIP-isolated PAO1 persisters (h) and CIP-isolated PW7144 persisters (i). Green arrows indicate OMVs.

While ERV was effective against multiple types of persister cells (CEFT, TOB & CCCP), we found it ineffective against fluoroquinolone (FQ) isolated persisters. Specifically, ciprofloxacin (CIP) and ofloxacin (OFX) isolated *P. aeruginosa* persister cells of PAO1 and PDO300 were not responsive to ERV (Figure 5a). To obtain more insights into the different effects between FQ persisters and other types of persisters tested, we followed the growth of FQ isolated persister cells with and without ERV treatment as we did for the other persisters shown in Figure 2. We are interested in FQs because they are commonly prescribed for *P. aeruginosa* infections. Our results demonstrate that CIP and OFX persisters resuscitated with detectable growth at approximately 20 h after extracellular ERV was removed (Figure 5b,c). This is consistent with the CFU results. Earlier research by Mok and Brynildsen^32^ found DNA damage in FQ-isolated *E. coli* persisters, and showed that DNA repair is required before growth can resume.^32^ This will likely reduce ERV efficiency by prolonging the delay in resuscitation, during which ERV may diffuse out before sufficient resumption of cellular activities that allows killing to occur. To test this hypothesis, we tested ERV against CIP-isolated *E. coli* persisters. Interestingly, ERV was found effective in killing CIP-persisters of *E. coli* (99.5 ± 9.0%; Figure S6), similar to the killing of ampicillin-persisters that we reported earlier.^18^ This finding indicates that the internal DNA damage in FQ persister cells might not be the primary reason that ERV is unable to kill CIP-persisters of *P. aeruginosa*. We thus investigated the penetration of ERV between CIP-isolated persisters and CCCP-isolated persisters of PAO1. We found that CIP-persisters only accumulated 6.2 ± 1.3 μg/mL of ERV, while CCCP-persisters accumulated 60.6 ± 8.0 μg/mL of ERV (Figure 5g) after treatment with 100 μg/mL ERV. The significant difference in ERV penetration helps explain the observed difference in persister killing by ERV.

Exposure to CIP has been shown to activate the SOS pathway in *P. aeruginosa* and enhance the production of outer membrane vesicles (OMVs).^33^ To understand if this can explain the difference in persister killing, scanning electron microscopy (SEM) was used to examine the phenotypic traits of different types of persister cells. The SEM images revealed small protruding circular structures on the membrane of CIP isolated persisters, which appeared to be OMVs (Figure 5d). The same structures were not observed on the membranes of either CCCP-isolated persisters or stationary phase cells (Figure 5d). To further evaluate if OMVs have adverse effects on ERV against CIP persisters, a mutant strain with suppressed SOS pathway was challenged with ERV following the same persister killing assay described above. Specifically, we used a *recA* mutant strain (PW7144) as it is known that *RecA* gets activated during DNA damage, which subsequently activates the SOS pathway. Thus, the *recA* mutant produces less OMVs compared to the wild-type strain.^33,34^ At the concentration of 100 μg/mL, ERV exhibited stronger killing activity against CIP-isolated PW7144 persisters (77.5 ± 7.9% killing; *p* < 0.0001) compared to the wild-type cells (no significant killing). This suggests that the SOS pathway and OMV production may play a role in the observed difference between CIP-isolated persisters and other types of persisters (Figure 5e). In addition, a higher intracellular concentration of ERV was detected in the CIP-isolated PW7144 persisters than the wild-type control (47.0 ± 2.9 vs 6.2 ± 1.3 μg/mL) after treatment at 100 μg/mL. To understand if this occurs to other FQs, we tested ERV on PW7144 persisters isolated with another FQ antibiotic, ofloxacin (OFX). The results revealed that OFX isolated PW7144 persister cells also had more killing (51.6 ± 5.1% killing) compared to wild-type PAO1 (*p* = 0.0012, Figure 5a&e). Consistently, no OMVs were found associated with PW7144 persisters based on SEM analysis (Figure 5f). TEM analysis further validated the appearance of OMVs on CIP-isolated PAO1 persisters, which were absent on CIP-isolated PW7144 persisters (Figure 5h,i). Taken together, these results demonstrate that OMVs may affect ERV penetration and/or accumulation in FQ isolated persisters of *P. aeruginosa*. This could hinder ERV from reaching its target site. Further studies on how antibiotics traffic around the OMVs will help the design of new persister control agents.

## Discussion

Persister cells are highly tolerant to antibiotics and play an important role in chronic infections.^30,35^ Our recent study on *E. coli* persisters^18^ described a new approach to persister control by targeting persister membrane penetration. The present study further validated that ERV can also effectively target and kill *P. aeruginosa* persisters cells, including those within biofilms and those involved in lung infection in a murine model. Although persister cells do not have the cellular activities (e.g., protein synthesis) for killing to occur, it still has permeability to antibiotics that do not require active transport to penetrate bacterial membranes and bind to the target. Antibiotics like ERV that are capable of penetrating persister cells and binding with the target site strongly can kill persister cells during wakeup. This provides useful hints to identify leads for persister control. Furthermore, our animal study provided encouraging evidence that *in vivo* condition allows for wakeup and thus persister killing by internalized ERV. This demonstrates a promising approach that can overcome the challenges of persister control with conventional antibiotics. Because conventional antibiotics were discovered based on growth inhibition, they are generally ineffective against dormant persister cells.^36^ Identifying new leads that can penetrate both planktonic and biofilm-associated persister cells under *in vivo* conditions deserves further research.

Studying the effects of ERV on persister cells specifically *in vivo* is challenging because persister cells are transient phenotypic variants. Currently, there is no established animal model to test persister killing directly because inoculation generates a mixture of normal and persister cells that can switch back and forth based on the environment. Previous studies have used chronic infection models or localized infection models such as murine epicutaneous infection model with *Staphylococcus aureus*,^37^ mouse deep-seated thigh infection model with MRSA,^38^*E. coli* O1_57_:H_7_ (EHEC) infection model within nematode *Caenorhabditis elegans*,^39^ and acute/chronic pulmonary infection using *P. aeruginosa*.^40^ While these models allow for the study of antibiotics in complex living systems, which include both actively growing cells and dormant bacterial populations, they do not provide specific testing conditions on persister cells directly. Therefore, in this study, we pretreated persister cells to evaluate the effects of internalized ERV *in vivo* during lung infection. This design enabled us to determine if the environment within the lungs will allow persister cells to wake up and thus become more susceptible to ERV that is accumulated within these cells. The results showed that internalized ERV can effectively execute its bactericidal action during wakeup *in vivo*. This finding provided more insights into how ERV could potentially be used for controlling persistent infections.

The results also demonstrated the possibility of combination therapy, in which ERV can work synergistically with other antibiotics to enhance treatment efficacy and reduce the burden of chronic infections. Combination therapy is an effective approach to eradicating persistent infections.^13,41−44^ Others have indicated that this approach can enable differential killing of a heterogeneous population and potentially prevent the development of antimicrobial resistance from surviving persister cells.^45^ Previously, we reported a strategy of sequential treatment with two antibiotics, which is effective in eradicating persister cells.^18^ The first antibiotic is used to eliminate active cells followed by a second antibiotic that targets the persister population specifically. Consistent results were reported by other studies,^13,41−43,46,47^ where the first antibiotic eliminates cells that have high metabolic activities, and the second antibiotic targets less active cells. This combination can kill both metabolically active cells and dormant cells such as persisters.^13^ Similarly, eradicating *Borrelia burgdorferi*, the causative agent of Lyme disease, requires three antibiotics, including doxycycline, cefoperazone, and daptomycin.^42^ The combination of all three antibiotics led to effective killing of the persister population. Another study combined the use of CIP and colistin to eliminate persister cells of *P. aeruginosa*,^46^ while the combination of CIP and vancomycin reduced the number of persister cells of *S. aureus*.^47^ In our study, we observed the effects of ERV on persister cells isolated with different antibiotics. The combination of ERV with CEFT, TOB, or CCCP all led to effective killing of *P. aeruginosa* persister cells.

Overall, this study further demonstrates the potential of persister control by selecting the right agent to penetrate persister membranes and achieve killing during wakeup. Specifically, we report that ERV is a potent agent against *P. aeruginosa* persister cells including persister cells in biofilms. In addition, we report that the killing by ERV during persister wakeup can occur *in vivo* in a murine lung infection model. The results provided important new information that wakeup process *in vivo* does provide the required window to achieve killing before ERV diffuses out. Further studies are needed to establish an animal model that can control persister formation *in vivo* for testing under more clinically relevant conditions. This is part of our ongoing work.

## Methods

### Bacterial Strains and Growth Media

All strains and conditions are listed in Supporting Information.

### ERV Treatment of Stationary/Exponential Phase *P. aeruginosa* Cells

Overnight cultures of *P. aeruginosa* PAO1 and PDO300 were grown for 16 h with inoculation from a single colony. After culturing, the cells were harvested by centrifugation, washed, and resuspended in PBS and then immediately treated with different concentrations (10–100 μg/mL) of ERV (MedChemExpress, NJ, USA) for 1 h at 37 °C with shaking at 200 rpm. After treatment, the cells were washed once with PBS to remove extracellular ERV molecules, and then plated to determine viability using the drop plate method.^48^ Three biological repeats were tested for each condition. For treatment of exponential phase cells, overnight cultures of PAO1 and PDO300 were subcultured in LB with a starting OD_600_ of 0.05 and incubated until OD_600_ reached 0.3–0.45. Cells were collected by centrifugation, resuspended in 500 μL PBS (OD_600_ = 0.5), washed three times with PBS (pH 7.4), and then proceeded to ERV treatment as described above. Details about the checkerboard assay and determination of combination index are listed in Supporting Information.

### Persister Isolation Using Different Antibiotics

The conditions for persister isolation, treatment and resuscitation can be found in Supporting Information.

### Biofilm Growth

*P. aeruginosa* biofilms were grown on PDMS (polydimethylsiloxane) surfaces for 48 h. After washing, the biofilms were treated with different concentrations of ERV. The viability of biofilm cells was determined by CFU assay or Live/Dead staining. Details of PDMS surface preparation, biofilm formation, and analysis can be found in Supporting Information.

### Animal Study

Original FVB/N mice were purchased from Jackson Laboratory (Bar Harbor, ME, USA). Male and female mice that were 8 to 12 weeks old (weight ∼ 25 g) were used in this study. Mice were maintained under specific pathogen-free conditions in the animal core facility at SUNY Upstate Medical University. The animal study was conducted in accordance with the Protocol Number IACUC 380B approved by the Institutional Animal Care and Use Committee of SUNY Upstate Medical University. FVB/N mice were injected with ∼10^7^ of untreated CCCP-isolated PAO1 persister cells, TOB treated CCCP isolated PAO1 persister cells, or ERV treated CCCP-isolated PAO1 persister cells (all in 50 μL of saline solution) intratracheally into the lung to induce infection.^49^ Preparation of the cells was conducted the same way as described above. Total cell numbers were determined using a hemocytometer and CFU assay. At 24 h post treatment, bronchoalveolar lavage fluid (BALF) was collected and analyzed to determine bacterial viability. More details can be found in Supporting Information.

### Scanning Electron Microscopy and Transmission Electron Microscopy Analyses

To observe the phenotypic characteristics of different types of persister cells, scanning electron microscopy (SEM) and transmission electron microscopy (TEM) were used. Details of sample preparation for both SEM and TEM can be found in Supporting Information.

### Statistical Analysis

All data were analyzed using one-way or two-way ANOVA followed by Tukey test if not noted otherwise using SAS version 9.13 (SAS Institute, Cary, NC, USA) or PRISM GraphPad Prism version 10.0.2 (GraphPad Software, Boston, Massachusetts USA). Differences with *p* < 0.05 were considered statistically significant.

## Supporting Information Available

Additional experimental details; time and dose dependent killing to determine persister isolation conditions; persister counts after isolation with different antibiotics; PAE after ERV treatment; standard curves of the reporter strain; effects of CIP on *E. coli* persisters, and intracellular concertation of ERV in *P. aeruginosa* biofilm cells (PDF)
