# Supplementary material for: Eradication of Pseudomonas aeruginosa Persister Cells by Eravacycline
Source: ACS Infect Dis. 2024 Nov 13;10(12):4127–36. doi: 10.1021/acsinfecdis.4c00349 (PMC11650763; doi:10.1021/acsinfecdis.4c00349)
Supplement: Supplementary file 1 — id4c00349_si_001.pdf [file id4c00349_si_001.pdf]

Supporting Information

Eradication of *Pseudomonas aeruginosa* persister cells by eravacycline

Sweta Roy[1], Zeynep S. Cakmak[1], Salma Mahmoud[2], Mahsa Sadeghzadeh[2],

Guirong Wang[2,3,*] and Dacheng Ren[1, 4, 5,6,*]

[1]Department of Biomedical and Chemical Engineering, Syracuse University, Syracuse, NY 13244, United States; [2]Department of Surgery, SUNY Upstate Medical University, Syracuse, NY 13210, United States; [3]Department of Microbiology and Immunology, SUNY Upstate Medical University, Syracuse, NY 13210, United States; [4]BionInspired Institute, Syracuse University, NY 13244, United States; [5]Department of Civil and Environmental Engineering, Syracuse University, Syracuse, NY 13244, United States; [6]Department of Biology, Syracuse University, Syracuse, NY 13244, United States

***Corresponding authors:**

Dacheng Ren: Phone +1-315-443-1257. Fax +1-315-443-9175.

Email: dren@syr.edu

Guirong Wang: Phone +1-315-464-6283; Fax +1-315-464-6236.

Email: Wangg@upstate.edu

Table of Contents

1. Materials and Methods……………………………………………………………………..S1
2. Supplementary Figures and Table…………………………………………………………….S4
3. References……………………………………………………………………………………….S10

Materials and Methods. This section lists additional details about materials and experimental methods that supplement the main text.

Bacterial strains and growth media. *Pseudomonas aeruginosa* PAO1,[1] its isogenic mutant PDO300 (*mucA22*),[2] and *S. aureus* ALC2085[3] were routinely cultured in Lysogeny broth (LB) containing 10 g/L NaCl, 5 g/L yeast extract, and 10 g/L tryptone. PW7144 (*recA*-359::IS*lacZ*/*hah*)[4] was cultured in LB with 20 μg/mL of tetracycline. *E. coli* HM22[5] (AT984 *dapA* zde-264::Tn10 *hipA7*) was cultured in LB supplemented with 25 μg/mL diaminopimelic acid (DPA).[5]

ERV treatment of stationary phase *P. aeruginosa* cells. *P. aeruginosa* PAO1 and PDO300 were grown for 16 h with inoculation from a single colony. The cells were then harvested by centrifugation, washed, resuspended in PBS to a final density of OD_{600} of 1.0, and then immediately treated with different concentrations (10 – 100 μg/mL) of ERV (MedChemExpress, NJ, USA) for 1 h at 37°C with shaking at 200 rpm. The effective concentration range is higher than the minimum inhibitory concentration (MIC), which is 6 μg/mL for both PAO1 and PDO300. This is expected for persister cells.[6] After treatment, extracellular antibiotic was removed by centrifugation (3 mins at 13,000 rpm) and the cells were washed once with PBS. To determine viability, cells were plated on LB agar plates to count colony forming units (CFU) using the drop plate method.[7]

ERV treatment of exponential phase *P. aeruginosa* cells. Overnight cultures of PAO1 and PDO300 were sub-cultured in LB with a starting OD_{600} of 0.05 and incubated until OD_{600} reached 0.3-0.45. Then the cells were collected by centrifugation (13,000 rpm, 3 mins) at RT. The OD_{600} was adjusted to 0.5 in 500 μL PBS. The cells were then washed three times with PBS (pH 7.4) and proceeded to ERV treatment as described above.

Checkerboard assay. To determine if CCCP played a role in ERV killing of persister cells, a checkerboard assay was conducted. Briefly, 16 h overnight cultures of PAO1 and PDO300 were treated with increasing concentrations of CCCP (0 – 400 μg/mL) in LB medium with a starting OD_{600} of 0.2 in a v-bottom 96-well plate. The cells were incubated for 3.5 h at 37°C with shaking at 200 rpm. After incubation, the cells were pelleted by centrifugation at 4,150 rpm for 3 mins and washed three times with PBS using a Biotek 50 TS Microplate Washer (BioTek, Winooski, VT, USA). The resuspended cells with PBS were then treated with increasing concentrations of ERV (0 – 200 μg/mL) for 1 h in PBS. Treatment in PBS allows the study on persister cells specifically (no growth factor added). To determine viability after treatment, the treated cells were pelleted by centrifugation, washed once with PBS and plated on LB agar plates to count CFU.

Combination index. The combination index classifies if the interaction between drug molecules is synergistic, additive, or antagonistic. Overnight cultures (16 h) of PAO1 and PDO300 were treated with increasing concentrations of CCCP (0, 10, 30, 50 & 100 μg/mL), ERV (0, 10, 30, 50 & 100 μg/mL) or combination of both CCCP and ERV (0, 10, 30, 50 & 100 μg/mL for each drug) in LB medium with a starting OD_{600} of 1.0. The cells were treated for 3.5 h with shaking at 200 rpm at 37°C. After treatment, the cells were collected by centrifugation (3 mins at 13,000 rpm) and washed with PBS to remove free antibiotic in the solution. The cells were then resuspended in PBS and plated on LB agar plates to count CFU using the drop plate method.[7] The combination index was calculated based on previously reported formula: $CI = (C_{A,x}/IC_{x,A}) + (C_{B,x}/IC_{x,B})$, where CI is the combination index value; $C_{A,x}$ and $C_{B,x}$ are the combined antibiotic drug concentrations needed when x percentage of the population is killed; $IC_{x,A}$ and $IC_{x,B}$ represent the individual drug concentrations needed when x percentage of the population is killed.[8]

Persister isolation using different antibiotics. To determine the conditions needed for persister isolation using different antibiotics, both the time-kill and dose-kill curves were generated for both PAO1 (Figure S1) and PDO300 (Figure S2). Fluoroquinolones such as CIP and OFX are effective against both growing

and non-growing bacteria. In comparison, the effect of CEFT and TOB depend on the growth phase of *P. aeruginosa*. To isolate persister cells, the concentration and time were determined based on biphasic killing curves (Figure S1 and S2).

(a) Persister isolation using CEFT and TOB. Overnight cultures (16 h) of PAO1 or PDO300 were sub-cultured in LB medium with a starting density of OD_{600} 0.05 and incubated until culture reached mid-exponential phase (OD_{600} reached 0.3-0.45). The cells were treated with either 100 µg/mL of CEFT or 60 µg/mL of TOB for 4 h at 37°C with shaking at 200 rpm. After isolation, extracellular antibiotic was removed by washing the cells three times with PBS before ERV treatment as described above.

(b) Persister isolation using CCCP, CIP and OFX. Overnight cultures (16 h) of PAO1, PDO300, or PW7144 were treated with either 200 µg/mL of CCCP[9] in LB, CIP in PBS or OFX in PBS with a starting OD_{600} of 1.0 for 3.5 h, 3.5 h, or 8 h, respectively, with shaking at 200 rpm at 37°C. After isolation, extracellular antibiotic was removed by washing the cells three times with PBS before ERV treatment as described above.

Resuscitation Assay. To observe the regrowth of the persister cells after the ERV was removed from the environment, persister cells were washed and resuspended in LB (500 µL). Then, 200 µL were transferred to a 96 well plate and incubated at 37°C with shaking at 200 rpm. Growth was monitored based on OD_{600} for 40 h using an Epoch 2 Microplate Spectrophotometer (BioTek, Winooski, VT, USA). PAE was calculated as PAE = T_{50}-C_{50}, where T_{50} and C_{50} are the time (in h) taken to reach 50% of the final optical density reached by an untreated control, for treated and untreated samples, respectively.[10, 11]

PDMS surface preparation. Biofilms were grown on PDMS (polydimethylsiloxane) surfaces. PDMS was prepared with 10:1 mixing of base : curing agent from the Sylgard® 184 elastomer kit.[12, 13] The mixture was vacuumed in a 50 mL falcon tube, poured onto a sterile 25 cm petri plate, and cured at 60°C for 24 h. After curing, PDMS surfaces of 10 mm × 0.5 mm were cut out using a sterile razor. The PDMS surfaces were then sterilized under UV light (30 mins for each side) before use.

Biofilm growth. *P. aeruginosa* biofilms (48 h) were formed on PDMS surfaces. 16 h culture of either PAO1 or PDO300 was used to inoculate a biofilm culture in 5 mL LB with a starting OD_{600} of 0.05 in a petri dish containing PDMS surfaces. The biofilm culture was grown for 48 h at 37°C without shaking. After 48 h, each PDMS surface with attached biofilm was washed three times with 0.85% NaCl solution and transferred to a new petri dish with PBS supplemented with ERV at 0, 10, 50, or 100 µg/mL. The biofilms were treated for 1 h at 37°C and viability was determined by CFU assay or imaging, adapted from a previously published protocol.[14] Briefly, each treated PDMS surface was transferred to a 5 mL polystyrene tube containing 1 mL PBS. The tubes containing the treated samples were gently sonicated for 1 min using a Branson Tabletop Ultrasonic Bath (Model B200, Branson Ultrasonics, Brookfield, CT, USA) and vortexed for 30 s to detach biofilm cells from the PDMS surface. The solution in the tube was collected and used to determine biofilm viability based on CFU. Detached cells during antibiotic treatment were also collected for biofilm viability assay based on CFU. The reported biofilm viability includes both cells detached during treatment and the cells that remained on the PDMS surface during antibiotic treatment. For image analysis, each PDMS surface was stained using LIVE/DEAD BacLight bacterial viability kit (Life Technologies Inc., Carlsbad, CA, USA). Biofilms were imaged using an Axio Imager M1 fluorescence microscope (Carl Zeiss Inc., Berlin, Germany). After deconvolution, the 3D fluorescence images were used to calculate biofilm biomass using COMSTAT[15-17].

Quantification of persister cells in treated biofilms. To determine the viable persister count within each biofilm after ERV treatment, the detached cells from ERV treated biofilms were treated with 60 µg/mL TOB in LB for 4 h at 37°C. After treatment, the viability was determined by CFU counts as described above.

Regrowth of treated biofilms. To determine if persister killing occurs in treated biofilms during wakeup, the cells from the ERV treated biofilms were incubated in LB medium for 2 h. Then the biofilm viability was determined based on CFU counts.

Quantification of intracellular concentration of ERV in biofilm cells. The intracellular concentration of ERV was determined using our previously described method.[12] Briefly, the reporter strain, *S. aureus* ALC2085, was treated with PAO1 cell lysate spiked with known concentrations of ERV to generate a standard curve (Figure S5), which was then used to determine the concentration of ERV in unknown samples. Briefly, the detached biofilm cells were pelleted down and lysed with chloroform. After removing cell debris by centrifugation (5,000 g, 5 mins), the sample was kept in a desiccator under vacuum overnight to remove residual solvent. On the following day, samples were dissolved in 100 μL sterile PBS (pH 7.4) with vertexing for 5 mins and used to treat the reporter strain as described above. The total cell number was determined using a hemocytometer for each biofilm condition. Antibiotic concentration was then quantified by fitting the standard curve (Figure S5).

BALF collection and cytology analysis. The animal test was conducted as described in the main manuscript in accordance with the Protocol Number IACUC 380B approved by the Institutional Animal Care and Use Committee of SUNY Upstate Medical University. Bacteria were injected intratracheally into the lung to induce infection.[18] At 24 h post treatment, the lungs of each mouse were lavaged using sterile saline (3 x 0.5 mL) as described previously.[18] The bronchoalveolar lavage fluid (BALF) was then centrifuged at 250×g for 10 mins. Suspension was collected and bacterial counts were determined based on CFU. The pellet was resuspended in 1 mL of sterile saline and 200 μL was mounted onto a slide by cytospin centrifuge (Model ROTOFIX32 A, Hettich, Beverly, Massachusetts, USA) at 1000 rpm for 3 mins. The slides were then air-dried and stained with Hema-3 Stain Kit (Fisher Scientific, Kalamazoo, MI, USA). The samples were examined with an Eclipse TE2000-U research microscope (Nikon, Melville, NY, USA). The intracellular concentrations of ERV were determined as described above using the same reporter strain.

SEM Analysis. To observe the phenotypic characteristics of different types of persister cells, scanning electron microscopy (SEM) was used. Persister cells were isolated as described above. The collected cell pellet was fixed with 2.5% glutaraldehyde and 4.0% paraformaldehyde in PBS. One hundred microliters of the resuspended cells were then fixed onto small UV sterilized glass and incubated for 1 h at room temperature. The samples were washed three times with PBS, post-fixed with 2.0% osmium tetroxide for 1 h, and then washed again. The samples were dehydrated in a graded ethanol series (30%, 50% 70% 90%, and then 100%) for 10 min each. The final dehydration in 100% ethanol was repeated three times. Samples were critical point dried (CO_2) and coated with a platinum sputter (Edwards S150A, Edwards, Burgess Hill, UK) under 20 mA with 30 s deposition time and imaged at an accelerating voltage of 10-15 kV using a JEOL JSM-IT 100LA SEM (JEOL Ltd., Tokyo, Japan). MountainsSEM® was used to add color to the SEM images.

TEM Analysis. To visualize the OMVs, CIP PAO1 persisters and CIP PW7144 persisters were fixed in 2.5% glutaraldehyde and 4% paraformaldehyde in 0.1 M cacodylate buffer pH = 7.2 overnight at 4°C. Samples were washed in cacodylate buffer three times and stained with 2% osmium tetroxide in DI water for 2 h at room temperature. Samples were then washed with DI water and each pellet was embedded with 50 μL of warm 4% agarose. Once solidified, the samples were dehydrated through a graded series of ethanol (25, 50, 75, 95%, absolute ethanol twice) and two cycles of acetone, 10 min each. Samples were embedded in resin (EMbed 812 kit, Electron Microscopy Sciences, Hatfield, Pennsylvania, USA) and polymerized for 24 h at 60°C. After polymerization, 70 nm sections were cut with a diamond knife on Leica UC7 Ultramicrotome. The sections on copper grids were contrasted with uranyl acetate. Images were collected at SUNY Upstate Medical University TEM core facility on a JEOL JEM-1400 transmission electron microscope equipped with Gatan Orius SC1000 CCD camera (JEOL Ltd., Tokyo, Japan).

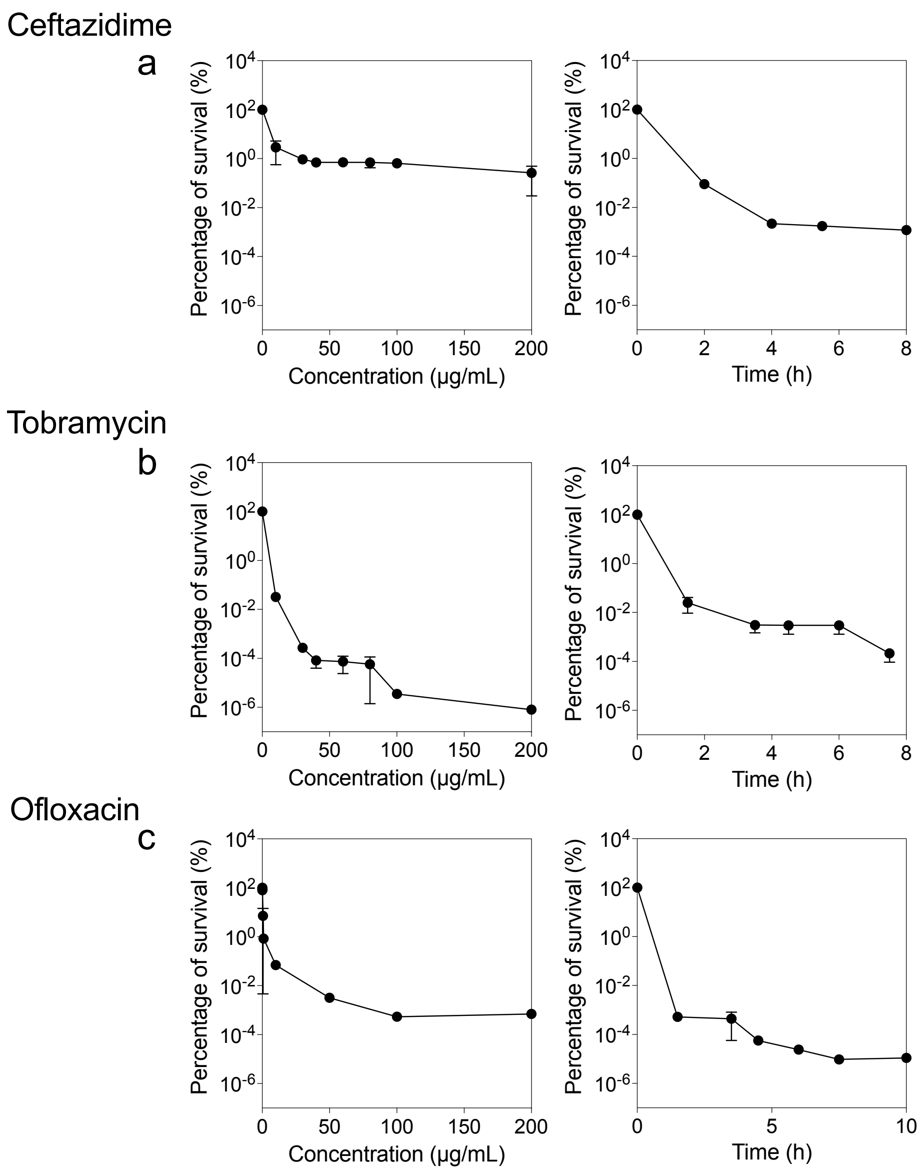

Figure S1. **Time and dose dependent killing to determine PAO1 persister isolation condition. (a)** PAO1 was grown to exponential phase and treated with different concentrations of ceftazidime (CEFT) for 3.5 h (left) or 100 µg/mL of CEFT for a total of 8 h (right). (**b)** PAO1 was grown to exponential phase and treated with different concentrations of tobramycin (TOB) for 3.5 h (left) or 60 µg/mL of TOB for a total of 8 h (right). **(c)** Stationary-phase PAO1 treated with different concentrations of ofloxacin (OFX) for 8 h (left) or 200 µg/mL of OFX for a total of 10 h (right).

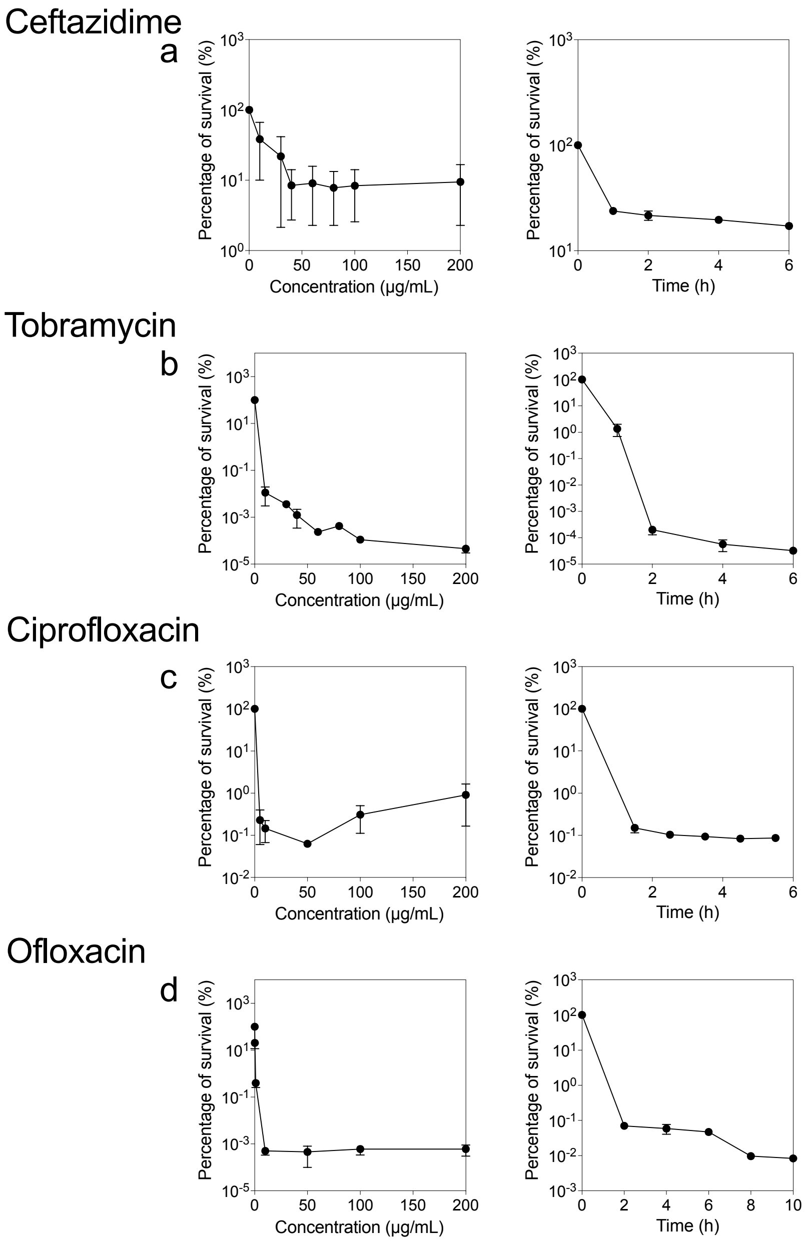

Figure S2. Time and dose dependent killing to determine PDO300 persister isolation condition. (a) PDO300 was grown to exponential phase and treated with different concentrations of ceftazidime (CEFT) for 3.5 h (left) or 100 μg/mL of CEFT for a total of 8 h (right). (**b)** PDO300 was grown to exponential phase and treated with different concentrations of tobramycin (TOB) for 3.5 h (left) or 60 μg/mL of TOB for a total of 8 h (right). **(c)** Stationary PDO300 treated with different concentrations of ciprofloxacin (CIP) for 3.5 h (left) or 200 μg/mL of CIP for a total of 6 h (right). **(d)** Stationary PDO300 treated with different concentrations of ofloxacin (OFX) for 8 h (left) or 200 μg/mL of OFX for a total of 10 h (right).

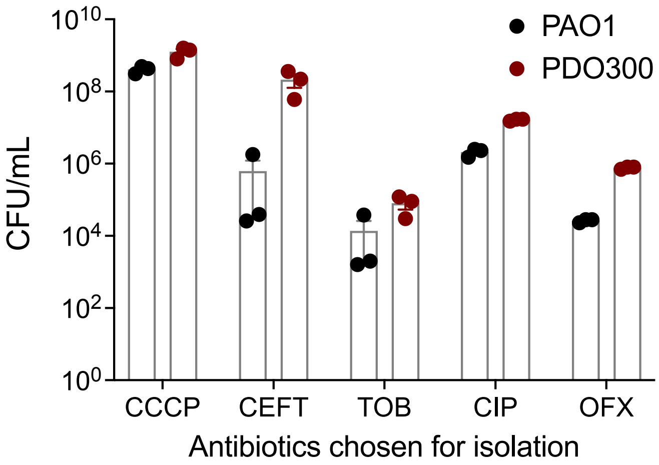

Figure S3. Persister counts. Number of PAO1 (black dots) and PDO300 (red dots) persister cells after isolation with different antibiotics (CCCP, CEFT, TOB, CIP & OFX). Means ± SE are shown (n=3).

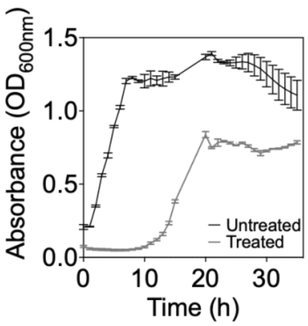

	C_{50}	T_{50}	PAE
Normal cells	4.0 ± 0.0	19.3 ± 0.3	15.3 ± 0.3
CCCP persisters	11.0 ± 0.0	ND	>60
CEFT persisters	5.3 ± 1.1	ND	>60
TOB persisters	21.3 ± 0.3	ND	>60
CIP persisters	30.3 ± 1.9	17.7 ± 1.4	-12.6 ± 2.8
OFX persisters	18.7 ± 0.3	37.0 ± 2.4	18.3 ± 2.2

Figure S4. Post-antibiotic effect (PAE) of PAO1 persisters after ERV treatment. (top) Exponential phase PAO1 cells were treated with 100 µg/mL of ERV for 1 h in PBS. After treatment, cells were washed and resuspended in LB medium. Growth was monitored through absorbance (OD_{600nm}). (bottom) Post-antibiotic effect (PAE) of PAO1 persisters and normal cells after ERV treatment. The results show resusitation after ERV treatement (1 h in PBS) for normal cells and persiter cells isolated with CCCP, CEFT, TOB, CIP, or OFX. Results from Figs. 2 & 5 were used to calculate PAE. For treated samples that did not reach 50% of the final absorbance of the untreated control (CCCP, CEFT & TOB), the T50 was labeled as undetectable (ND).

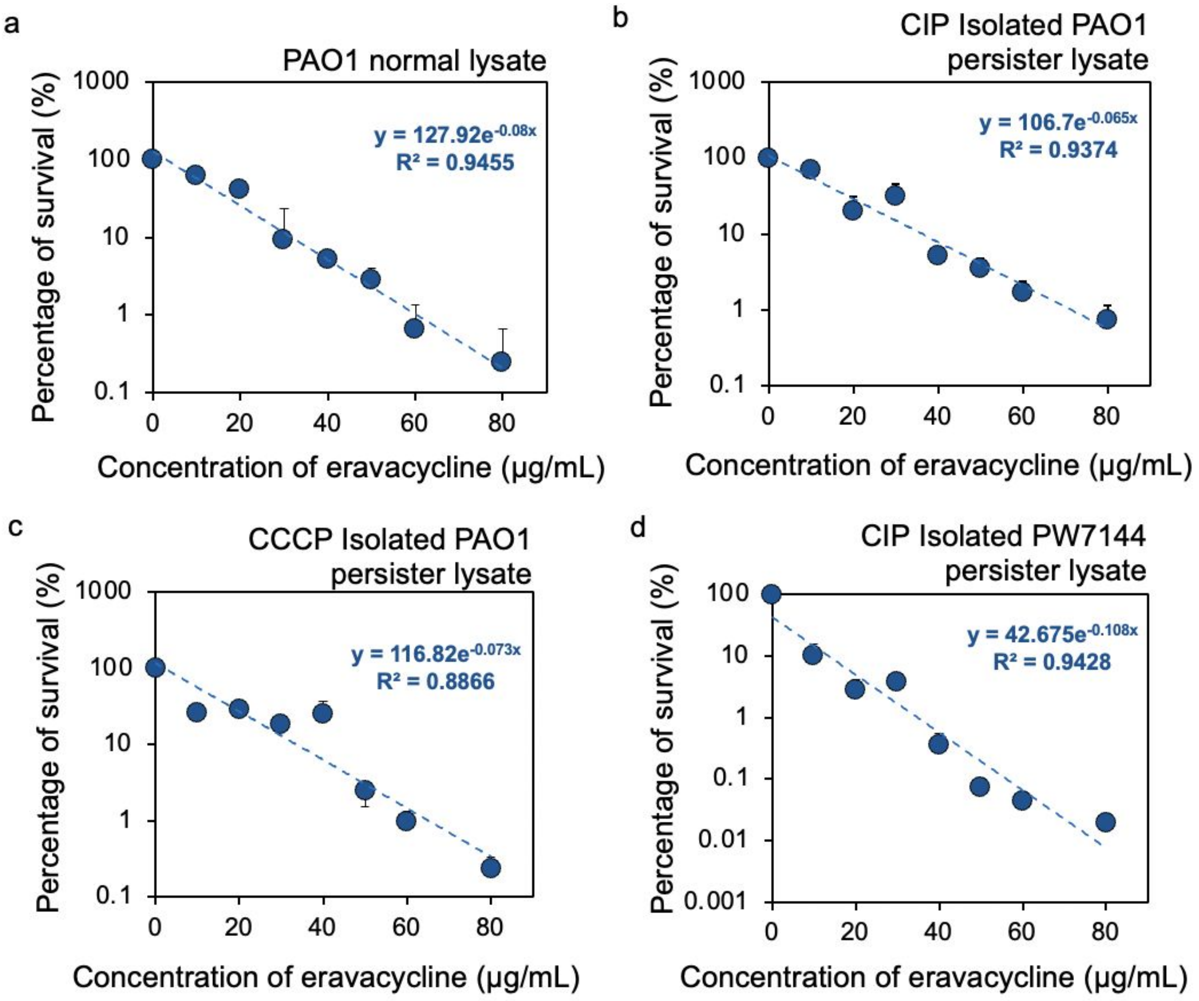

Figure S5. **Standard curves of reporter killing by ERV**. The standard curves of ERV were generated using the reporter strain *S. aureus* ALC2085 treated with either PAO1 normal cell lysates **(a)**, CIP PAO1 persister lysates **(b)**, CCCP PAO1 persister lysate **(c),** or CIP PW7144 persister lysates **(d),** supplemented with known concentrations of ERV. Means ± SE are shown (n=2).

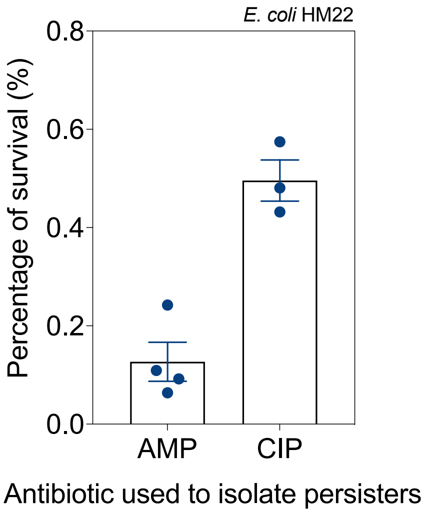

Figure S6. Viability of *E. coli* HM22 persisters. CIP isolated *E. coli* HM22 persister cells were treated with 100 µg/mL of ERV and compared to AMP isolated *E. coli* HM22 persister cells.

Table S1. Intracellular concentration of ERV in biofilm cells. To further understand the effects of ERV, 48 h PAO1 or PDO300 biofilms were treated with 100 µg/mL ERV. Intracellular concentration of ERV was quantified from both the attached cells (remained after treatment) and cells that detached during ERV treatment. Means ± SE are shown (n=2).

PAO1

Targeted Population	ERV concentration after per cell 100 µg/mL treatment
Attached cells	21.6 ± 1.9 µg/mL
Cells detached during treatment	23.6 ± 2.0 µg/mL

PDO300

Targeted Population	ERV concentration per cell after 100 µg/mL treatment
Attached cells	27.5 ± 6.0 µg/mL
Cells detached during treatment	33.5 ± 0.7 µg/mL

References

(1) Holloway, B. W. Genetics of *Pseudomonas*. *Bacteriol Rev* **1969**, *33* (3), 419-443. DOI: 10.1128/br.33.3.419-443.1969.

(2) Mathee, K.; Ciofu, O.; Sternberg, C.; Lindum, P. W.; Campbell, J. I. A.; Jensen, P.; Johnsen, A. H.; Givskov, M.; Ohman, D. E.; Søren, M.; et al. Mucoid conversion of Pseudomonas aeruginosa by hydrogen peroxide: a mechanism for virulence activation in the cystic fibrosis lung. *Microbiology (Reading)* **1999**, *145 (Pt 6)*, 1349-1357. DOI: 10.1099/13500872-145-6-1349.

(3) Sauer, K.; Steczko, J.; Ash, S. R. Effect of a solution containing citrate/Methylene Blue/parabens on *Staphylococcus aureus* bacteria and biofilm, and comparison with various heparin solutions. *J Antimicrob Chemother* **2009**, *63* (5), 937-945. DOI: 10.1093/jac/dkp060.

(4) Held, K.; Ramage, E.; Jacobs, M.; Gallagher, L.; Manoil, C. Sequence-verified two-allele transposon mutant library for *Pseudomonas aeruginosa* PAO1. *J Bacteriol* **2012**, *194* (23), 6387-6389. DOI: 10.1128/JB.01479-12.

(5) Keren, I.; Shah, D.; Spoering, A.; Kaldalu, N.; Lewis, K. Specialized persister cells and the mechanism of multidrug tolerance in Escherichia coli. *J Bacteriol* **2004**, *186* (24), 8172-8180. DOI: 10.1128/JB.186.24.8172-8180.2004.

(6) Jia, J.; Zheng, M.; Zhang, C.; Li, B.; Lu, C.; Bai, Y.; Tong, Q.; Hang, X.; Ge, Y.; Zeng, L.; Zhao, M.; Song, F.; Zhang, H.; Zhang, L.; Hong, K.; Bi, H. Killing of *Staphylococcus aureus* persisters by a multitarget natural product chrysomycin A. *Sci Adv* **2023**, *9* (31), eadg5995. DOI: 10.1126/sciadv.adg5995.

(7) Sieuwerts, S.; de Bok, F. A.; Mols, E.; de vos, W. M.; Vlieg, J. E. A simple and fast method for determining colony forming units. *Lett Appl Microbiol* **2008**, *47* (4), 275-278. DOI: 10.1111/j.1472-765X.2008.02417.x.

(8) Zhao, L.; Au, J. L.; Wientjes, M. G. Comparison of methods for evaluating drug-drug interaction. *Front Biosci (Elite Ed)* **2010**, *2*, 241-249. DOI: 10.2741/e86.

(9) Narayanaswamy, V. P.; Keagy, L. L.; Duris, K.; Wiesmann, W.; Loughran, A. J.; Townsend, S. M.; Baker, S. Novel Glycopolymer Eradicates Antibiotic- and CCCP-Induced Persister Cells in. *Front Microbiol* **2018**, *9*, 1724. DOI: 10.3389/fmicb.2018.01724.

(10) Stubbings, W. J.; Bostock, J. M.; Ingham, E.; Chopra, I. Assessment of a microplate method for determining the post-antibiotic effect in Staphylococcus aureus and *Escherichia coli*. *J Antimicrob Chemother* **2004**, *54* (1), 139-143. DOI: 10.1093/jac/dkh275.

(11) Rázquin-Olazarán, I.; Shahrour, H.; Martínez-de-Tejada, G. A synthetic peptide sensitizes multi-drug resistant *Pseudomonas aeruginosa* to antibiotics for more than two hours and permeabilizes its envelope for twenty hours. *J Biomed Sci* **2020**, *27* (1), 85. DOI: 10.1186/s12929-020-00678-3.

(12) Roy, S.; Bahar, A. A.; Gu, H.; Nangia, S.; Sauer, K.; Ren, D. Persister control by leveraging dormancy associated reduction of antibiotic efflux. *PLoS Pathog* **2021**, *17* (12), e1010144. DOI: 10.1371/journal.ppat.1010144.

(13) Roy, S. A New Strategy for Persister Control. Syracuse University, *PhD Dissertations - ALL*. 1692. https://surface.syr.edu/etd/1692, 2023.

(14) Wang, H.; Tampio, A. J. F.; Xu, Y.; Nicholas, B. D.; Ren, D. Noninvasive Control of Bacterial Biofilms by Wireless Electrostimulation. *ACS Biomater Sci Eng* **2020**, *6* (1), 727-738. DOI: 10.1021/acsbiomaterials.9b01199.

(15) Heydorn, A.; Nielsen, A. T.; Hentzer, M.; Sternberg, C.; Givskov, M.; Ersbøll, B. K.; Molin, S. Quantification of biofilm structures by the novel computer program COMSTAT. *Microbiology (Reading)* **2000**, *146 (Pt 10)*, 2395-2407. DOI: 10.1099/00221287-146-10-2395.

(16) *Comstat v. 2.1*. www.comstat.dk

(17) Vorregaard, M. Comstat2 - a modern 3D image analysis environment for biofilms in Informatics and Mathematical Modelling. In *Comstat2 - a modern 3D image analysis environment for biofilms in Informatics and Mathematical Modelling*, Technical University of Denmark: Kongens Lyngby, Denmark: Technical University of Denmark: Kongens Lyngby, Denmark, 2008.

(18) Yang, F.; Zhang, J.; Yang, Y.; Ruan, F.; Chen, X.; Guo, J.; Abdel-Razek, O.; Zuo, Y. Y.; Wang, G. Regulatory Roles of Human Surfactant Protein B Variants on Genetic Susceptibility to *Pseudomonas Aeruginosa* Pneumonia-Induced Sepsis. *Shock* **2020**, *54* (4), 507-519. DOI: 10.1097/SHK.0000000000001494.
